# Real-World Effectiveness of Glucose-Guided Eating Using the Data-Driven Fasting App Among Adults Interested in Weight and Glucose Management: Observational Study

**DOI:** 10.2196/65368

**Published:** 2025-05-08

**Authors:** Michelle R Jospe, Martin Kendall, Susan M Schembre, Melyssa Roy

**Affiliations:** 1 Georgetown Lombardi Cancer Center, Georgetown University, 3800 Reservoir Rd NW, Washington, DC, 20007, United States, 1 202-444-2223; 2 Optimising Nutrition LLC, Brisbane, Australia; 3 Department of Medicine, University of Otago, Dunedin, New Zealand

**Keywords:** precision health, digital health, metabolic health, personalized nutrition, blood glucose self-monitoring, biological feedback, glucose, blood glucose, monitoring, self monitoring, dietary intervention, diet, app engagement, glucose monitoring

## Abstract

**Background:**

The Data-Driven Fasting (DDF) app implements glucose-guided eating (GGE), an innovative dietary intervention that encourages individuals to eat when their glucose level, measured via glucometer or continuous glucose monitor, falls below a personalized threshold to improve metabolic health. Clinical trials using GGE, facilitated by paper logging of glucose and hunger symptoms, have shown promising results.

**Objective:**

This study aimed to describe user demographics, app engagement, adherence to glucose monitoring, and the resulting impact on weight and glucose levels.

**Methods:**

Data from 6197 users who logged at least 2 days of preprandial glucose readings were analyzed over their first 30 days of app use. App engagement and changes in body weight and fasting glucose levels by baseline weight and diabetes status were examined. Users rated their preprandial hunger on a 5-point scale.

**Results:**

Participants used the app for a median of 19 (IQR 9-28) days, with a median of 7 (IQR 3-13) weight entries and 52 (IQR 25-82) glucose entries. On days when the app was used, it was used a median of 1.8 (IQR 1.4-2.1) times. A significant inverse association was observed between perceived hunger and preprandial glucose concentrations, with hunger decreasing by 0.22 units for every 1 mmol/L increase in glucose (95% CI −0.23 to −0.21; *P*<.001). Last observation carried forward analysis resulted in weight loss of 0.7 (95% CI −0.8 to −0.6) kg in the normal weight category, 1 (95% CI −1.1 to −0.9) kg in the overweight category, and 1.2 (95% CI −1.3 to −1.1) kg in the obese category. All weight changes nearly doubled when analyzed using a per-protocol (completers) analysis. Fasting glucose levels increased by 0.11 (95% CI 0.09-0.12) mmol/L in the normal range and decreased by 0.14 (95% CI −0.16 to −0.12) mmol/L in the prediabetes range and by 0.5 (95% CI −0.58 to −0.42) mmol/L in the diabetes range. Per-protocol analysis showed fasting glucose reductions of 0.26 (SD 4.7) mg/dL in the prediabetes range and 0.94 (16.9) mg/dL in the diabetes range.

**Conclusions:**

The implementation of GGE through the DDF app in a real-world setting led to consistent weight loss across all weight categories and significant improvements in fasting glucose levels for users with prediabetes and diabetes. This study underscores the potential of the GGE to facilitate improved metabolic health.

## Introduction

Self-monitoring glucose levels before eating can help people recognize their physiological cues to eat, improve glycemic control, and help modify eating behaviors. This method has previously been referred to as hunger recognition [1] and hunger training [2], and is now termed glucose-guided eating (GGE) [3,4]. Early clinical trials confirmed that self-monitoring of glucose could be a feasible and effective approach to promote weight loss and insulin sensitivity in adults without diabetes [2,5,6]. Other subsequent trials of GGE in similar populations of adults without diabetes have demonstrated equally intriguing effects on weight loss, improved insulin resistance, and reduced glycemic variability [3,4,7]. Despite the promise, little is known about the real-world effectiveness and adherence to GGE.

GGE is designed as a short-term, intensive training program to help individuals develop long-lasting self-regulation skills in response to physiological hunger. By encouraging users to eat only when preprandial glucose levels are near or below a personalized threshold, GGE aims to enhance mindful eating habits, improve metabolic health, and reduce reliance on external cues for eating. Hunger is a natural physiological response that drives eating behavior, and GGE uses near-fasting glucose levels as a personalized proxy for hunger, reflecting short-term energy availability. Clinical trials implementing GGE typically involve a structured training period of up to 30 days, during which participants monitor their preprandial glucose levels using portable glucometers or wearable continuous glucose monitors. Previous research has demonstrated that eating when glucose is low can reduce glycemic variability, fasting insulin, and Homeostatic Model Assessment of Insulin Resistance (HOMA-IR) [3,4], with these improvements potentially lowering the risk of chronic diseases such as cardiovascular disease and certain cancers [8,9]. This initial training period has also been shown to result in benefits that persist for months beyond the structured intervention [10].

GGE also has a psychological and behavioral basis. Research has shown that eating when glucose is below a specified threshold helps people distinguish between eating driven by physiological hunger and eating driven by hedonic, emotional, or habitual factors [11]. Our previous results have shown that following GGE can reduce emotional eating, increase awareness of hunger and satiety, reduce the number of eating occasions, and help individuals explore the effects of different types of foods on their glucose levels [7,12].

The principles of GGE have been incorporated into Data-Driven Fasting (DDF; Optimising Nutrition LLC), an app that has been commercially available since 2021 [13]. Similar to clinical trials of GGE, DDF helps users learn to eat in response to physiological hunger by self-monitoring their preprandial glucose. Users are encouraged to eat only when their preprandial glucose levels are below their personalized threshold, as described by GGE [2]. However, the method for establishing personalized glucose thresholds in DDF differs from traditional GGE applications. Traditional GGE typically uses average morning fasting glucose levels to set a single static threshold that remains constant throughout the experience. In contrast, DDF uses preprandial glucose levels and continuously updates these thresholds throughout the challenge period. DDF also provides nutritional recommendations based on pre- and postprandial glucose levels to improve the glycemic quality of the diet and limit postprandial hyperglycemia at subsequent meals, an approach not previously used in GGE. Furthermore, DDF offers flexibility in adhering to glucose thresholds by providing an alternative threshold for times when users struggle to stay below their established threshold. In addition, self-monitoring of glucose in research settings has historically been done with paper-and-pen logs, whereas DDF uses a mobile app that features progress tracking and educational resources.

The increasing availability of glucose monitoring technology enhances the relevance and potential impact of DDF. Glucometers are readily available to the general population, and the accessibility of continuous glucose monitors is broadening, particularly with the release of over-the-counter models in several countries. Glucose monitoring technology provides valuable and immediate biological feedback about short-term energy availability and the metabolic consequence of carbohydrate intake. Consequently, it represents a feasible and practical intervention tool that can help people improve their metabolic health. While clinical trials are promising, it is unknown whether GGE, when facilitated by a commercially available mobile app and implemented in a free-living environment, can be adhered to or produce clinically meaningful effects on weight loss or improvements in blood glucose. Therefore, the goal of this study is to describe the demographic characteristics of DDF users, their engagement with the app and adherence to preprandial glucose monitoring, and the subsequent impact on weight and glucose levels, providing insights into the real-world implementation of GGE and its potential to improve metabolic health outcomes.

## Methods

### Study Design

This was an observational study of DDF app users from January 1, 2021, to December 8, 2023. Participants in this study were not recruited but were existing users of the commercially available DDF app. Data were collected from users who voluntarily engaged with the app as part of their normal use, and all data analyzed were deidentified entries made during the first 30 days of app use.

### DDF App

**Figure 1.**
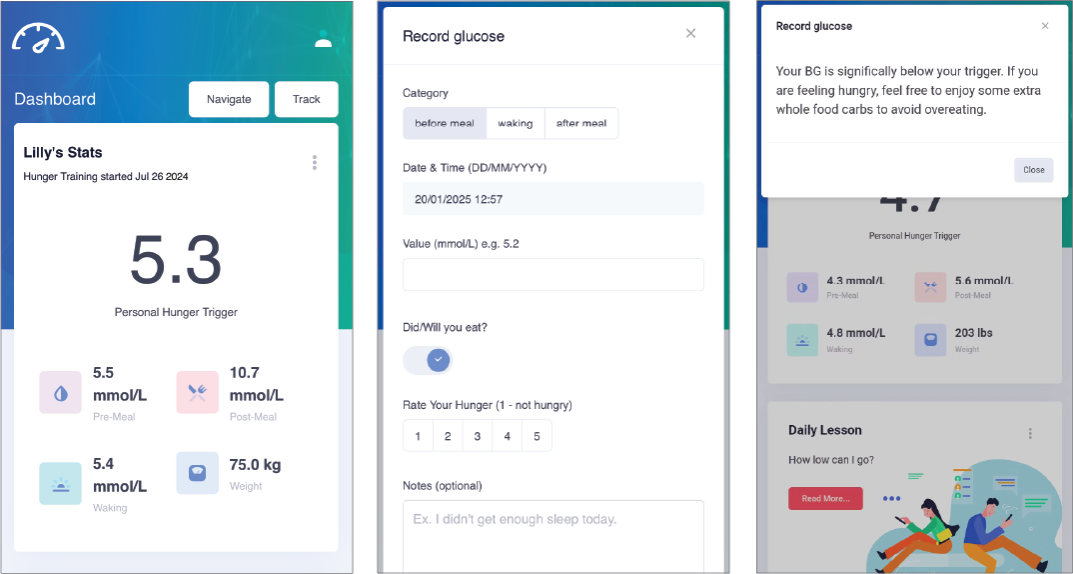
Screenshots of the Data-Driven Fasting web-based app.

DDF is a freely accessible web-based app with a subscription option [13]. Users could pay a small fee for a structured 30-day program that includes daily lessons, weekly live question and answer sessions, and community support. Users are encouraged to purchase a glucometer for glucose tracking, with the Contour Next (Ascensia Diabetes Care) being the recommended model. DDF has an interactive interface designed to help users follow GGE and provides nutritional guidance based on recorded preprandial (premeal) and postprandial (postmeal) self-monitored glucose levels. Perceived preprandial hunger ratings were recorded in the app using a 5-point scale, with 1 indicating “not hungry” (Figure 1).

### DDF Initiation

During the first 3 days of app use, participants are instructed to eat normally but to test their glucose levels before every eating occasion. Reported preprandial glucose levels during this time are averaged to establish a baseline glucose threshold. After this initial phase, users are encouraged to observe their hunger sensations and test their preprandial glucose levels only when hungry, and to delay eating until their glucose levels fall below their personalized threshold (ie, glucose-guided eating). After 5 days of glucose-guided eating, the threshold is adjusted daily by averaging reported preprandial glucose over the previous 7 days, with a maximum decrease of 0.5% per day. During times marked by higher-than-usual glucose levels due to conditions other than recent eating (eg, high stress, fatigue, pain, and intense exercise) users can opt to use an alternative, higher threshold (+0.6mmol/L or +10 mg/dL above their current threshold).

### DDF Nutritional Guidance

The DDF app provides automated nutritional guidance based on recorded preprandial and postprandial glucose levels (relative to the current glucose threshold). When preprandial glucose is slightly above the threshold (≤0.4mmol/L or 7mg/dL), users are encouraged to prioritize meals with a higher protein composition. When glucose is just below the threshold, users are encouraged to eat “a nutritious meal.” If preprandial glucose is considerably below the threshold (>0.4mmol/L or 7mg/dL), users are encouraged to prioritize “whole food carbs” to quickly raise glucose into a normal range. Users who choose to also report postprandial glucose levels also receive feedback. Specifically, in the event postprandial glucose levels are more than 1.6 mmol/L or 30 mg/dL above the current threshold, the app suggests they “consider eating less of it (or even avoiding) that meal in the future.”

### Outcome Measures

In addition to monitoring pre- and postprandial glucose levels and perceived hunger levels, the DDF app also allows users to track their fasting glucose levels and body weight. For this study, we extracted all available data entered into the DDP app by users during the first 30 days of use with day 0 representing the first day a preprandial glucose level was recorded. Implausible weight (<30 to >300 kg, and other unlikely values based on neighboring weights), height (<120 to >230 cm), and glucose values (<2.8 to >11.1 mmol/L) were excluded from the dataset.

### Statistical Analysis

In an effort to examine the effectiveness of the DDF app, we aimed to assess the changes in body weight (primary outcome) and fasting glucose levels (secondary outcome) between day 0 and day 30 of using the DDF app. The analysis included users who had at least 2 days of recorded entries. A total of 6519 people used the DDF app, with 95.1% (n=6197) using it at least twice in the first 30 days and thus were included in the analytical sample. To ensure the robustness of the findings, a sensitivity analysis was conducted to compare results when including users with 2 or more days of data versus 7 or more days. The findings were consistent across both criteria, confirming that the inclusion of users with fewer days of data did not affect the outcomes. Descriptive results, such as app usage data, were summarized using appropriate measures of central tendency (eg, means or medians) and variability (eg, SDs or IQRs), depending on the data distribution. Participants were categorized based on their baseline BMI and fasting glucose levels using established clinical thresholds [14,15]. Changes in weight and fasting glucose levels between the first and last weeks of app use were assessed using paired comparisons with 95% CIs. For participants who did not have weight or glucose measures in the last week, the last observation carried forward (LOCF) method was applied. A linear mixed-effects model was used to examine the relationship between preprandial glucose and perceived hunger, with preprandial glucose included as a fixed effect and a random intercept for participants to account for repeated measures within individuals. This approach allowed for the estimation of the overall association between preprandial glucose and hunger while accounting for variability in baseline hunger levels across users. Model estimates were reported with 95% CIs, and the significance of the fixed effect was assessed. Analyses were done with R (v4.2.2, R Foundation for Statistical Computing).

### Ethical Considerations

This study was approved by the University of Otago Human Ethics Committee (reference: HD22/064). Participants were users of a commercially available app who consented to their anonymized data being used for research purposes through the app’s privacy policy. All data were deidentified before analysis. No compensation was provided for participation.

## Results

On average, participants in the analytical sample had a BMI in the obese range (≥30 kg/m^2^), fasting glucose in the prediabetes range (5.6−6.9 mmol/L), and were predominantly female (Table 1). Participants primarily resided in North America (4590/6197, 74.1%), Europe (732/6197, 11.8%), and Australia and New Zealand (652/6197, 10.5%). No age data were collected, and height data were missing for 1152/6197 users (18.6%).

Those with only 1 day of entries (n=322) were excluded from the analysis and were similar to the analytical sample in terms of baseline weight (*P*=.08) and fasting glucose (*P*=.41) but had a lower BMI (24.5kg/m², *P*=.01) and included significantly more men and nonbinary individuals (*P*<.001).

**Figure 2.**
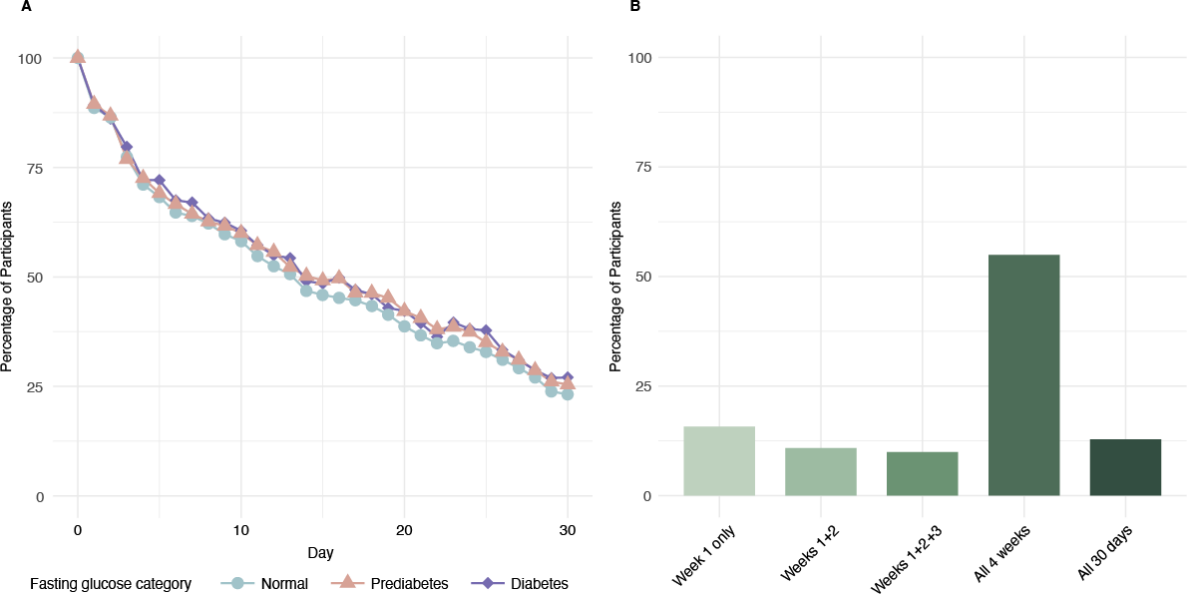
Data-Driven Fasting app engagement: (A) daily engagement by fasting glucose category and (B) weekly engagement.

The app was used by the analytical sample for a median of 19 (IQR 9-28) days, with 7 (IQR 3-13) weight entries and 52 (IQR 25-82) glucose entries, which were primarily preprandial glucose entries (Table 2). On days when the app was used, it was used a median of 1.8 (IQR 1.4-2.1) times. Using at least one preprandial glucose entry as a proxy for daily use, app engagement decreased from 100% at baseline to 47% by day 15% and 24% by day 30. Daily engagement patterns, broken down by fasting glucose category, exhibited similar trends, with consistent decreases across all groups. In contrast, weekly engagement trends show that the majority of users engaged with the app across all 4 weeks (Figure 2). Users rated their preprandial hunger on a 5-point scale. A significant inverse association was observed between perceived hunger and preprandial glucose concentrations, with hunger decreasing by 0.22 units for every 1 mmol/L increase in glucose (95% CI −0.23 to −0.21, *P*<.001). This effect corresponds to a negative linear relationship, analogous to an inverse Pearson correlation, while accounting for individual variability in baseline hunger levels (random intercept SD 0.63).

The analysis of weight changes for the total analytical sample and by baseline BMI category showed consistent reductions across all groups (Table 3). There were initially 4 users with a BMI in the underweight category, but none provided a weight in the last week of the intervention and were therefore excluded from the analysis. Using LOCF analysis, those with a BMI in the normal weight, overweight, and obese categories lost 0.7 kg, 1 kg, and 1.2 kg, respectively. All weight changes were nearly doubled when analyzed per protocol (completers) analysis (only considering those who provided weight measurements at week 0 and week 4).

**Figure 3.**
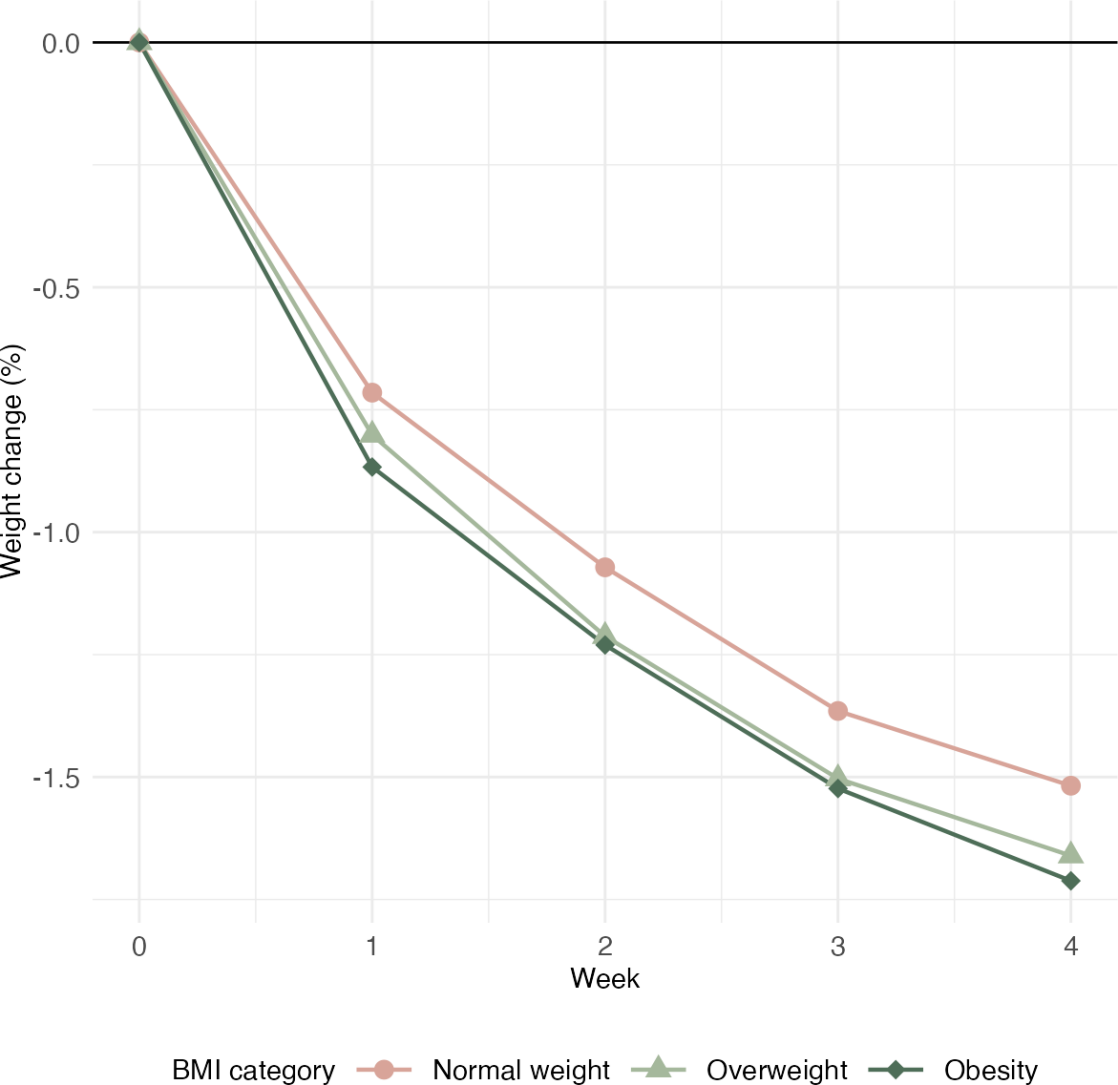
Weight trajectories by baseline BMI using last observation carried forward (n=3304).

Figure 3 shows the weight change by percentage of baseline weight using the last observation carried forward. Over 4 weeks, those with a BMI in the normal, overweight, and obese ranges lost 1.5% (95% CI 1.4%-1.6%), 1.7% (95% CI 1.6%-1.8%), and 1.7% (95% CI 1.6%-1.7%) of weight, respectively.

**Figure 4.**
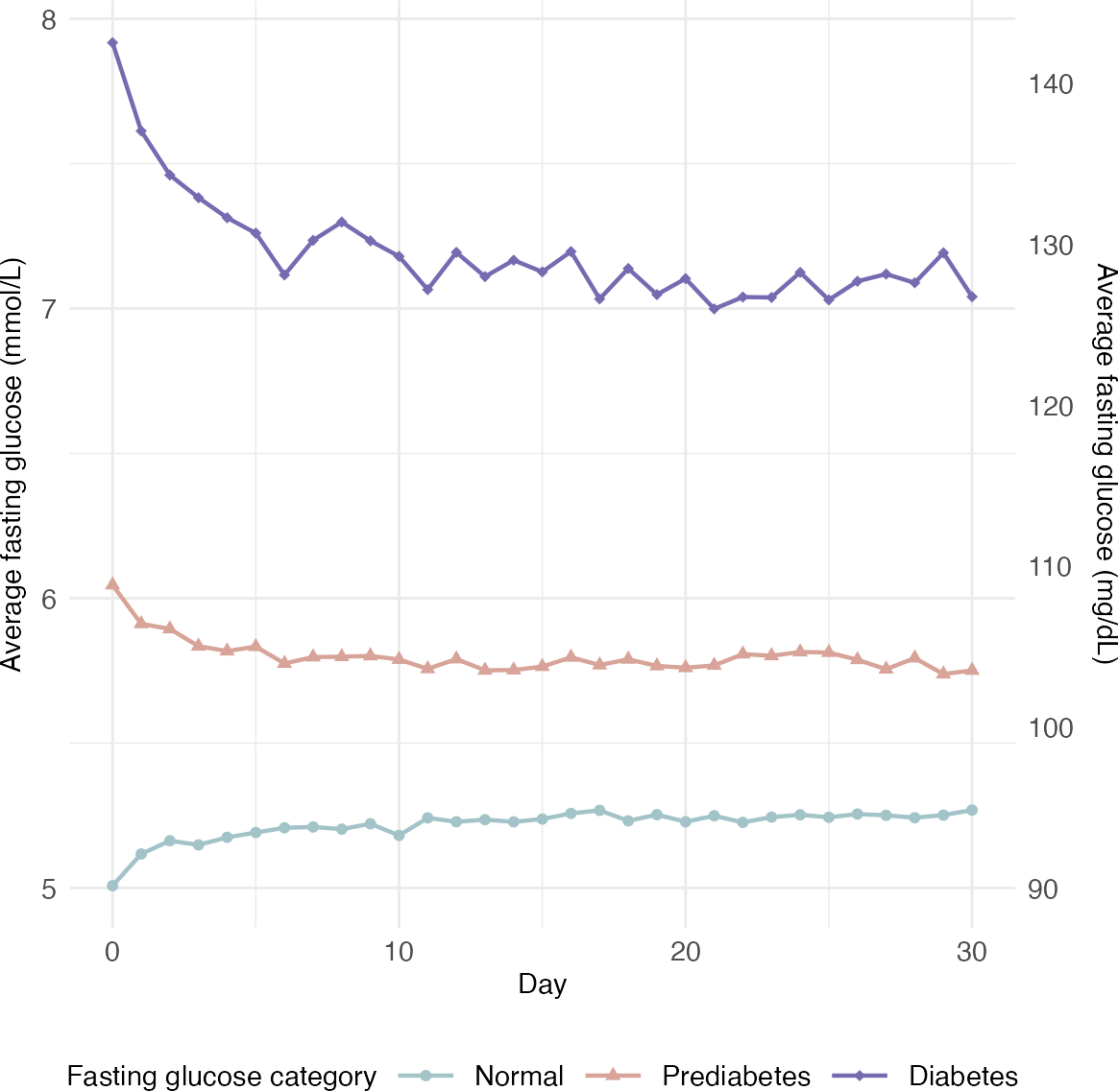
Fasting glucose trajectories by baseline fasting glucose (n=6092).

**Figure 5.**
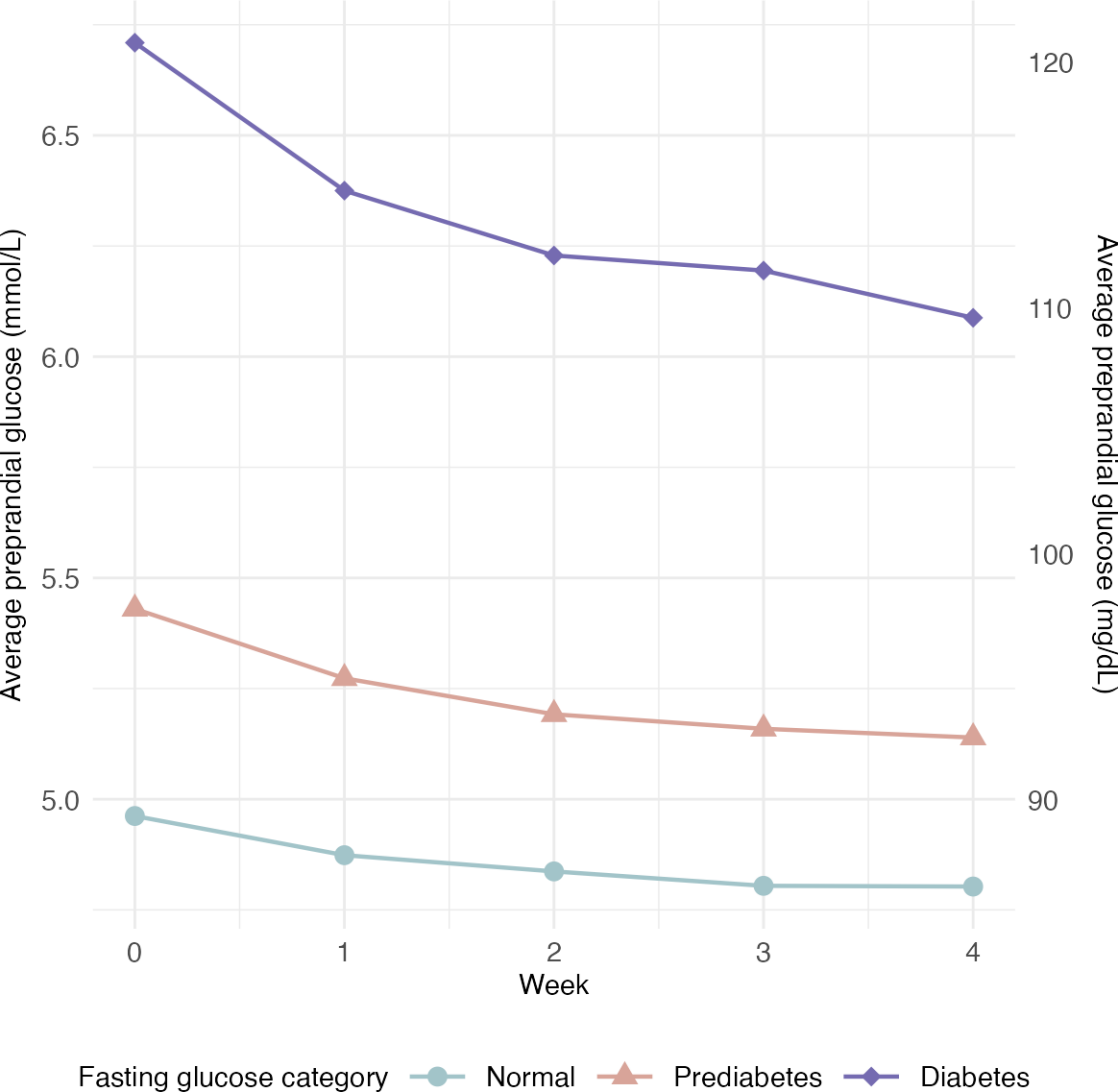
Preprandial glucose trajectories by baseline fasting glucose (n=6092).

The analysis of fasting glucose changes for the total analytical sample and by baseline fasting glucose category revealed significant changes across all groups (Table 4 and Figure 4). Using LOCF, those with fasting glucose levels in the prediabetes and diabetes ranges had a reduction in fasting glucose of 0.14 mmol/L (2.5 mg/dL) and 0.5 mmol/L (9 mg/dL), respectively. Like with weight changes, the difference in fasting glucose was nearly doubled when only considering those with fasting glucose data at week 0 and week 4, with those in the prediabetes and diabetes ranges reducing their fasting glucose by 0.26 mmol/L (4.7 mg/dL) and 0.94 mmol/L (16.9 mg/dL), respectively. Conversely, those with fasting glucose levels in the normal range had an increase in glucose of 0.11 mmol/L (2 mg/dL) with the LOCF analysis and 0.23 mmol/L (4.1 mg/dL) for those with fasting glucose values at week 0 and week 4. Preprandial glucose decreased for all fasting glucose categories (Figure 5). Over 4 weeks, those with a fasting glucose in the normal, prediabetes, and diabetes ranges had a mean reduction in preprandial glucose of 0.16 (95%CI 0.13-0.19) mmol/L, 0.29 (95% CI 0.25-0.33) mmol/L, and 0.62 (95% CI 0.45-0.8) mmol/L, respectively.

**Table 1.** Baseline characteristics of Data-Driven Fasting app users from January 2021 to December 2023 (n=6197).

Characteristics	Values	
Weight (kg) (n=4465), mean (SD)	83.9 (18.9)	
BMI (kg/m^2^) (n=3308), mean (SD)	30.1 (6.3)	
Underweight (<18), n (%)	4 (0.1)	
Normal weight (≥18->25), n (%)	690 (20.9)	
Overweight (≥25), n (%)	1162 (35.1)	
Obesity (≥30), n (%)	1452 (43.9)	
Fasting glucose (mmol/L) (n=6092), mean (SD)^a^	5.8 (1.1)	
Normal (≤5.5), n (%)	2723 (44.7)	
Prediabetes (5.6‐6.9), n (%)	2763 (45.4)	
Diabetes (≥7), n (%)	606 (10)	
Gender (n=6197), n (%)		
Women	5130 (82.8)	
Men	1052 (17)	
Nonbinary	15 (0.2)	

a Based on American Diabetes Association classifications.

**Table 2.** Data-Driven Fasting app engagement (n=6249).

Variable	Total number in 30-day period, median (IQR)
Days used	19 (9-28)
Weight entries	7 (3-13)
Glucose entries	52 (25-82)
Fasting glucose entries	11 (5-21)
Preprandial glucose entries	27 (12-43)
Postprandial glucose entries	10 (5-20)

**Table 3.** Weight changes by BMI category.

Participant categories	Week 0		Week 4		Per protocol (those with weight at week 0 and 4)		Last observation carried forward	
	n^a^	Weight (kg), mean (SD)	n^a^	Weight (kg), mean (SD)	n^a^	Weight change (kg), mean (95% CI)	n^a^	Weight change (kg), mean (95% CI)
All participants	3304	83.9 (18.9)	2102	81.7 (17.7)	2102	−2.2 (−2.3 to −2.1)	3304	−0.9 (−0.9 to −0.8)
Normal weight	690	64.3 (7.3)	365	63.3 (6.8)	365	−1.4 (−1.5 to −1.2)	690	−0.7 (−0.8 to −0.6)
Overweight	1162	77 (9.3)	635	75.4 (9)	635	−1.8 (−1.9 to −1.6)	1162	−1.0 (−1.1 to −0.9)
Obesity	1452	98.7 (16.3)	757	95.7 (15.6)	757	−2.3 (−2.4 to −2.1)	1452	−1.2 (−1.3 to −1.1)

a Number of participants.

**Table 4.** Fasting glucose changes by fasting glucose category.

Participant categories	Week 0		Week 4		Per protocol (those with glucose at week 0 and 4)		Last observation carried forward	
	n^a^	Fasting glucose, mean (SD)	n^a^	Fasting glucose, mean (SD)	n^a^	Fasting glucose change (mmol/L), mean (95% CI)	n^a^	Fasting glucose change (mmol/L), mean (95% CI)
All participants	6027	5.8 (1.1)	3066	5.7 (1)	3022	−0.13 (−0.16 to −04.1)	6092	−0.06 (−0.08 to −0.05)
Normal	2698	5 (0.4)	1282	5.3 (0.6)	1268	0.23 (0.19 to 0.26)	2723	0.11 (0.09 to 0.12)
Prediabetes	2739	6.1 (0.4)	1458	5.8 (0.7)	1441	−0.26 (−0.3 to −0.23)	2763	−0.14 (−0.16 to −0.12)
Diabetes	590	8.1 (1.1)	326	7.1 (1.5)	313	−0.94 (−1.07 to −0.81)	606	−0.50 (−0.58 to −0.42)

a Number of participants.

## Discussion

### Principal Findings

Previous research indicates that in experimental settings, GGE results in meaningful improvements in body weight, theory-based eating behaviors, and metabolic health outcomes, particularly those related to glucose metabolism. DDF, a commercially available mobile app that supports GGE, represents a scalable, personalized public health intervention with a growing evidence base. This study aimed to evaluate DDF app engagement and the effectiveness of DDF on changes in body weight and fasting glucose levels. The findings indicate that DDF produces modest improvements in body weight, particularly among users with higher weights at baseline, and clinically meaningful reductions in fasting glucose, particularly among users with fasting glucose in the diabetes range at baseline. These results support the effectiveness of DDF despite a steady decline in app use over the 30-day period.

DDF produced an average weight loss of 2.2 kg in the completer analysis, comparable with the results of studies using GGE in more controlled research settings of different durations. Average weight loss in the published clinical trials ranges from 1.5 kg in a 2-week feasibility trial for participants with overweight or obesity [2] to 4‐5 kg over 6 months [7,10]. Despite its simplicity and reliance on intermittent glucose monitoring, DDF achieved 30-day weight losses comparable with that reported in a study evaluating the January AI app, which uses continuous glucose monitoring and artificial intelligence–driven personalized dietary feedback [16]. This comparison underscores the effectiveness of GGE, which relies on intermittent preprandial glucose measurements and simpler personalized thresholds, in achieving comparable real-world weight loss. The modest inverse association between hunger and glucose observed in this study aligns closely with findings from previous work conducted with participants across a range of BMI categories [2]. Results from this commercial implementation of GGE are reassuring, confirming the real-world potential of this approach to improve body weight.

In contrast, this is the first GGE study to report a clinically meaningful effect on fasting glucose [17]. The results here are similar to a recent 8-week clinical trial of time-restricted eating in a sample of adults with obesity, which resulted in a reduction in fasting blood glucose of 0.4 mmol/L with greater improvements among participants who had elevated blood glucose levels at baseline [18]. Interestingly, among users who had normal fasting blood glucose at baseline, use of the DDF app resulted in a slight increase in fasting glucose levels over the 30-day period (0.11 mmol/L to 0.23 mmol/L or 2 mg/dL to 4 mg/dL), although still within the normal range (<5.6 mmol/L or <100 mg/dL). Future studies should aim to explore this phenomenon, if replicated. Despite subtle fluctuations of fasting glucose among normoglycemic users of the DDF app, the majority of users experienced clinically meaningful improvements in glycemia. Future clinical trials should aim to replicate the beneficial effects of GGE on markers of glucose metabolism (and insulin resistance), particularly among people with prediabetes or diabetes.

The strengths of this study include the real-world application of GGE and a very large sample size that included a diverse range of baseline weights and fasting glucose levels. The results demonstrate the outcomes of the DDF method when implemented under real-world conditions. Data collected from people in free-living conditions are more representative of effectiveness and can be better generalized to the wider population. The DDF app provides an accessible and structured method for glucose-guided eating, leveraging user-friendly tools like glucometers to promote self-awareness and behavioral changes.

Despite these strengths, there was a lack of available data describing users in terms of age, ethnicity, and health status; however, the app could be easily modified to collect these data for future research purposes. In addition, our analyses examining the effect of DDF on body weight and fasting glucose levels were limited by the use of self-reported data. Adherence to the program could only be inferred from interaction with the DDF app and the absence of granular tracking tools, such as Google Analytics, limits the ability to fully evaluate user interaction with the app. It is possible that users continued to follow GGE without recording all measures or used the app incorrectly. While the median app usage of 19 days reflects real-world engagement patterns, it may not capture the long-term impact of sustained use. However, previous research has demonstrated that the benefits of GGE can persist for up to 5 months following the initial training period [10]. The DDF app focuses on helping users establish effective self-regulation strategies within the first 30 days, which may contribute to lasting benefits. Future research should investigate the durability of these outcomes over extended periods. The study design did not include a control group, which limits our ability to definitively conclude that changes in our outcomes were due to the use of DDF. While the use of the glucometer alone (and their companion apps) might have induced added benefits, research to date suggests that these benefits are likely to be minor without a structured program [19,20]. Compared with gold-standard measures like laboratory fasting glucose or HbA_1c_, preprandial glucose monitoring with a glucometer offers a more accessible yet less precise approach [21]. This trade-off highlights the practicality of the DDF app for real-world settings, particularly for individuals seeking to manage weight and glucose levels without requiring expensive or complex equipment.

For users, the DDF app provides a simple and structured framework to improve metabolic health by promoting self-awareness of hunger and glucose patterns. For health care professionals, the app provides a unique opportunity to observe real-world glucose patterns and weight trends, offering insights that could inform personalized dietary and metabolic health interventions. Adherence and outcomes in the real world are likely to be affected by a range of external factors, such as social support, food security, family structure, and food preferences, which were not possible to measure in this setting. Nevertheless, these preliminary findings are positive, particularly in light of the real-world research setting, and suggest a more rigorous examination of the long-term potential benefit of DDF is warranted.

### Conclusions

GGE, supported by the DDF app, is a simple intervention that could be easily implemented at scale with minimal support services. Glucometers are widely available and inexpensive, and continuous glucose monitors are available over-the-counter in several countries. Results from this study suggest that the DDF app may be most beneficial for people with overweight or obesity and those who have dysglycemia. The DDF app could be used alongside other lifestyle interventions to support behavior change among these populations. Future randomized controlled trials are warranted to confirm the metabolic benefits of DDF and GGE in specific populations, such as individuals with prediabetes or diabetes, and to assess the sustainability of the behavioral and metabolic improvements achieved through the app over the long term.

## Abbreviations

DDF: Data-Driven Fasting
GGE: glucose-guided eating
HOMA-IR: Homeostatic Model Assessment of Insulin Resistance
LOCF: last observation carried forward
